# Ensemble-based methods for forecasting census in hospital units

**DOI:** 10.1186/1471-2288-13-67

**Published:** 2013-05-30

**Authors:** Devin C Koestler, Hernando Ombao, Jesse Bender

**Affiliations:** 1 Department of Community and Family Medicine, Geisel School of Medicine at Dartmouth College, Lebanon, NH 03756, USA; 2 Department of Statistics, University of California at Irvine, Irvine, CA 92697, USA; 3 Department of Pediatrics, Women and Infants Hospital of Rhode Island, Providence, RI 02905, USA

## Abstract

**Background:**

The ability to accurately forecast census counts in hospital departments has considerable implications for hospital resource allocation. In recent years several different methods have been proposed forecasting census counts, however many of these approaches do not use available patient-specific information.

**Methods:**

In this paper we present an ensemble-based methodology for forecasting the census under a framework that simultaneously incorporates both (*i*) arrival trends over time and (*ii*) patient-specific baseline and time-varying information. The proposed model for predicting census has three components, namely: current census count, number of daily arrivals and number of daily departures. To model the number of daily arrivals, we use a seasonality adjusted Poisson Autoregressive (PAR) model where the parameter estimates are obtained via conditional maximum likelihood. The number of daily departures is predicted by modeling the probability of departure from the census using logistic regression models that are adjusted for the amount of time spent in the census and incorporate both patient-specific baseline and time varying patient-specific covariate information. We illustrate our approach using neonatal intensive care unit (NICU) data collected at Women & Infants Hospital, Providence RI, which consists of 1001 consecutive NICU admissions between April 1st 2008 and March 31st 2009.

**Results:**

Our results demonstrate statistically significant improved prediction accuracy for 3, 5, and 7 day census forecasts and increased precision of our forecasting model compared to a forecasting approach that ignores patient-specific information.

**Conclusions:**

Forecasting models that utilize patient-specific baseline and time-varying information make the most of data typically available and have the capacity to substantially improve census forecasts.

## Background

In a period of heightened economic burden, efficient and effective allocation of hospital resources is an issue of principal importance. The ability to accurately forecast the number of patient arrivals, as well as predict census counts in hospital departments, have considerable implications for hospital resource allocation, both at the micro and macro level. For example, short term census forecasts have the potential to improve in-patient bed allocation, reduce diversions, better align hospital ancillary services, and reduce the incidence of over- and under-staffing [1]. More importantly, accurate census forecasts can inform scaling up of operations during high census periods, potentially leading to improved patient outcomes [2]. Since staffing levels in hospital units are driven by the census capacity as well as the acuity of in-unit patients, forecasting methods that incorporate both patient-level severity of illness (which may evolve considerably throughout their stay) and long-term census trends are necessary for informing accurate census predictions - this is a main highlight of this paper.

There have been several methodologies developed for forecasting arrival and census counts in various hospital departments [3-8]. Jones *et al.* (2008) evaluated the use of seasonal autoregressive integrated moving average (ARIMA), time series regression, exponential smoothing, and artificial neural network models to forecast daily patient volumes in emergency departments at three diverse hospital emergency departments. The time series methods considered in that analysis provided improved in-sample model goodness of fit assessed via mean absolute prediction error (MAPE) relative to a multiple linear regression approach, considered the benchmark model for forecasting emergency department patient volumes. Additionally, Sun *et al.* (2009) evaluated the use of autoregressive integrated moving average models, adjusted to incorporate various environmental variables, to forecast counts of daily patient attendances in the emergency department of an acute care regional general hospital. In addition to univariate time series approaches to forecasting emergency department patient volumes, multivariate time series models have also been utilized and have been shown to reliably forecast emergency department patient census.

The primary limitation of the above methods is that they do not incorporate patient-level information to make predictions. Here, we propose an ensemble-based method for short-term census forecasts under a framework that simultaneously incorporates (*i*) hospital unit arrival trends over time and (*ii*) patient-specific baseline and time-varying information. Such approaches represent the future of census forecasting as hospital departments around the country move toward more efficient methods for collecting and processing patient-level information upon admission and through the duration of stay. The proposed method is applied to neonatal intensive care unit (NICU) data collected at Women & Infants Hospital, Providence RI, which consists of 1001 consecutive NICU admissions between April 1st 2008 and March 31st 2009. In order to illustrate the potential for improved census forecasts that results from incorporating baseline and time-varying patient information, our proposed approach is compared to a forecasting method that ignores patient-specific information.

## Methods

To elucidate our methodology, we differentiate between arrival, departure, and census counts for the NICU. We define the arrival count on a particular day as the number of patients admitted to the NICU during a 24 hour period. Similarly, the departure count for a particular day is defined as the number of patients who depart the NICU as a result of a healthy discharge during a 24 hour period. By healthy discharge we refer to cases where a patient was discharged from the NICU as a result of adequate physiological health, as determined by clinical criteria. Lastly, we define the daily census count as the number of patients residing in the NICU at the end of the day (11:59pm).

Our approach to modeling the NICU census follows [9] where the NICU census *C*(*t*+*k*) at time (*t*+*k*) can be concisely expressed as a function of several different components. More specifically, *C*(*t*+*k*) where *k*≥1, is a function of:

1. *C*(*t*): NICU census at time (*t*).

2. {*A*(*t*+1),*A*(*t*+2),…*A*(*t*+*k*)}: number of arrivals on each successive day from time (*t*+1) up to time (*t*+*k*).

3. {*D*(*t*+1),*D*(*t*+2),…*D*(*t*+*k*)}: number of departures from the NICU census on each successive day from time (*t*+1) up to time (*t*+*k*).

The census at time (*t*+*k*) takes the form:

(1) $$C(t+k)=\underset{\text{observed}}{\underset{︸}{C(t)}}+\underset{\text{not observed}}{\underset{︸}{\overset{k}{\underset{i=1}{\sum }}A\left(t,+,i\right)-\overset{k}{\underset{i=1}{\sum }}D\left(t,+,i\right)}}$$

so that, the census at time (*t*+*k*) is equal to the census at time (*t*) plus the number of arrivals on each successive day from time (*t*+1) up to time (*t*+*k*), minus the number of departures on each successive day from time (*t*+1) up to time (*t*+*k*). We note that, at the current time (*t*), the only observable component in model (1) is *C*(*t*). As such, the predicted census at time (*t*+*k*), $\hat{C}(t+k)$, must contain predictions for the number of arrivals and departures on each successive day up to time (*t*+*k*) from time (*t*+1), $\hat{A}(t+i)$ and $\hat{D}(t+i),i=1,2,\ldots ,k$. Thus, our census forecasting model can be succinctly expressed as:

(2) $$\hat{C}(t+k)=C(t)+\overset{k}{\underset{i=1}{\sum }}\hat{A}(t+i)-\overset{k}{\underset{i=1}{\sum }}\hat{D}(t+i)$$

### Remark 1

One key assumption we make is that the number of arrivals *A*(*t*) is independent of the number of departures *D*(*t*) at time (*t*). In other words, the number of patients arriving in the NICU census at some time (*t*) does not provide information about the number of patients departing the NICU census at that same time. As will be described in further detail, the assumption of independence between arrivals and departures at some time (*t*) has important implications when computing the errors associated with our census predictions. In general, this is a sensible assumption when NICUs are operating below their maximum capacity, which was most often the case for the data presented here.

### Remark 2

We note that the number of departures *D*(*t*+*k*) at time (*t*+*k*) is not independent of the number of arrivals *A*(*t*+*k*−*i*) at time (*t*+*k*−*i*), where *i*=1,2,…. That is, there is an upper-bound on the number of departures at time (*t*+*k*) based on the cumulative number of arrivals from the preceding days.

### Remark 3

In (2), the estimate for the census at time (*t*+*k*) is given as a point estimate, which is subject to uncertainty given the error in the estimation of the number of arrivals and departures. Stochastic or “ensemble-based” forecasting is used to account for this uncertainty through the use of multiple forecasts created with an individual forecast model. For example, we generate an ensemble of predictions of *C*(*t*+*k*), denoted $\left\{{\hat{C}}^{\left(r\right)}(t+k),r=1,2,\ldots ,M\right\}$, where *r* represents a single realization and *M* is an upper-bound on the number of realizations, and summarize over the ensemble to obtain more accurate census forecasts. We describe this procedure in further detail in Section “Ensemble-based forecasting and prediction intervals for census forecasts”.

Since obtaining census forecasts at some time (*t*+*k*) is contingent upon predictions for the number of arrivals as well as the number of departures from (*t*+1) up to and including time (*t*+*k*), we describe our proposed methodology for predicting the number of arrivals and departures in the paragraphs that follow.

### Predicting the number of arrivals

We model daily arrival using the Poisson Autoregressive (PAR) model [10]. This choice is inspired by several studies that have demonstrated that daily arrival patterns in various hospital departments can be modeled as a Poisson process [11-13]. Moreover, this model incorporates the correlation between day-to-day arrival counts. The model specification is as follows: let {*A*(*t*),*t*=1,2,…*T*}, denote a time series of arrival counts. We define $F_{t}$ as any covariate information, including previous arrival counts, available to the observer up to time *t*. Under the PAR model, denote the conditional expected arrival to be $E[A(t)|F_{t}]=\mu_{t}$, *t*=1,2,…,*T*.

Thus, the conditional mean of the *p*^*th*^ order PAR model using a log-linear link function is given by:

(3) $$\log[\mu_{t}(\beta )]=\beta_{0}+\overset{p}{\underset{i=1}{\sum }}\beta_{i}A(t-i)$$

where ***β***=[*β*_0_,*β*_1_,…,*β*_*p*_]^*T*^ is a vector of autoregressive parameters and *A*(*t*−*i*) represents the number of arrivals at time (*t*−*i*), *i*=1,2,…,*p*. Since arrival counts in the NICU tend to contain a seasonal pattern, we generalize equation (3) to include seasonality effects:

(4) $$\begin{array}{l} \log[\mu_{t}(\beta ,\phi )]=\beta_{0}+\overset{K}{\underset{k=1}{\sum }}\phi_{k}\cos\left(\frac{2\mathrm{\pi t}\omega_{k}}{T}\right)\\ \;+\alpha_{k}\sin\left(\frac{2\mathrm{\pi t}\omega_{k}}{T}\right)+\overset{p}{\underset{i=1}{\sum }}\beta_{i}A(t-i) \end{array}$$

Here, *ω*_*k*_ represents the frequency, equivalently *T*/*ω*_*k*_ is the period or the number of time points (days) needed to complete one cycle and *ϕ*_*k*_ and *α*_*k*_ capture the amplitude of the *k*^*th*^ seasonality effect. To estimate the seasonality components, we examined the cyclical properties of the autocorrelation function (ACF). This approach is consistent with the fact that the spectrum - which gives a frequency decomposition of the variance in the time series - is the Fourier transform of the autocovariance (or autocorrelation) sequence. In our data, a visual inspection of the sample ACF showed that there is a cosinusuidal pattern with half-year periodicity (one cycle every 26 weeks). To select the order (*p*) of the above model, we use the Bayesian Information Criterion (BIC) [14]. After determining the optimal order for the above model, which we call $\dot{p}$, we estimate the expected number of arrivals ${\hat{\mu }}_{t}$, via:

(5) $${\hat{\mu }}_{t}=\exp\left[{\hat{\beta }}_{0}+\hat{\phi }\cos\left(\frac{2\mathrm{\pi t\omega}}{T}\right)+\overset{\dot{p}}{\underset{i=1}{\sum }}{\hat{\beta }}_{i}A\left(t,-,i\right)\right]$$

where $\hat{\beta }$ and $\hat{\phi }$ denote the conditional maximum likelihood estimates for ***β*** and *ϕ* respectively, where we treat the first $\dot{p}$ arrivals, $\left\{A(1),A(2),\ldots ,A(\dot{p})\right\}$ as fixed.

### Predicting the number of departures

To predict the number of departures from a group of patients residing in the NICU, it is ideal to incorporate both patient-specific baseline covariate information (i.e., information collected upon admission to the NICU) and any covariate information collected throughout their stay in the NICU. For the data considered here, only birth weight and gestational age were obtained for each child upon NICU admission. Although both are known to be useful for predicting length of stay, physiologic information collected throughout their time in the NICU may dictate when patients are released and thus, largely impact overall length of stay [15]. In addition, we also seek a framework for predicting the number of departures to reflect the fact that the probability of a patient leaving *k* days from some time point (*t*) should be conditional on how many days that patient has already spent in the NICU.

#### Some notation

**Figure 1:**
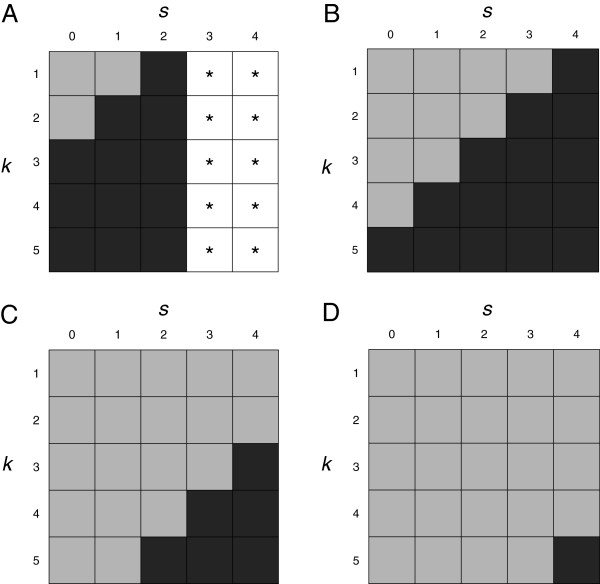
**Illustration of *Y***^***(k,s)***^** coding scheme.** Illustration of *Y*^(*k*,*s*)^ coding scheme at a fixed time *t* for a subject with (**A**) LOS = 3, (**B**) LOS = 5, (**C**) LOS = 7, and (**D**) LOS = 9. Black boxes represent *Y*^(*k*,*s*)^ = 1, grey boxes denote *Y*^(*k*,*s*)^ = 0, and boxes with ∗ represent cases where *Y*^(*k*,*s*)^ is undefined. In general, an observation is undefined if LOS ≤*s*.

Suppose *S*_*i*_ represents the number of days the *i*^*th*^ patient has spent in the NICU and **X**_*i*_ represents a vector of baseline covariates collected on the *i*^*th*^ patient. Moreover, define $X_{i}^{\left(t\right)}$ as a vector of additional covariates obtained up to and including (*t*) days after admission to the NICU for the *i*^*th*^ patient. Thus $Z_{i}^{\left(t\right)}={\left[X,X^{\left(t\right)}\right]}_{i}$, is a vector of baseline covariates plus any additional covariate information that is obtained for the *i*^*th*^ patient up to and including (*t*) days after admission to the NICU. Further, let *H*_*s*_(*t*) represent the number of patients in the NICU at time (*t*) who have stayed (*s*) days in the NICU and suppose that $Y_{i}^{\left(k,s\right)}$ is an indicator of whether or not patient *i* leaves the NICU in *k* days, given that they have already spent (*s*) days in the NICU. More specifically:

$$\;Y_{i}^{\left(k,s\right)}=\left\{\begin{array}{cc} 1 & \text{if}\;\text{LO}S_{i}\leq k+s,\text{given}\;(s)\text{days spent in the NICU}\\ 0 & \text{if}\;\text{LO}S_{i}>k+s,\text{given}\;(s)\text{days spent in the NICU} \end{array}\right)$$

where *LOS*_*i*_ represents the length of stay in the NICU for patient *i*. For example, when *k*=1 and *s*=0, we have the following,

$$\;Y_{i}^{\left(1,0\right)}=\left\{\begin{array}{cc} 1 & \text{if}\;\text{LO}S_{i}\leq 1\text{given (0) days spent in the NICU}\\ 0 & \text{if}\;\text{LO}S_{i}>1\text{given (0) days spent in the NICU} \end{array}\right)$$

which indicates whether or not patient *i* leaves the NICU prior to or on 1 day after their admission to the NICU. When *k*=1 and *s*=1, $Y_{i}^{\left(1,1\right)}$ indicates whether or not patient *i* leaves the NICU in 1 day after having spent 1 day in the NICU. Figure 1 further illustrates the *Y*^(*k*,*s*)^ coding scheme.

#### Modeling the probability of departure

Let $\pi^{\left(k\right)}(Z_{i}^{\left(s\right)},S_{i}=s)=P(Y_{i}^{\left(k,s\right)}=1|Z_{i}^{\left(s\right)},S_{i}=s)$, which represents, at time (*t*), the probability that patient *i*, who has spent (*s*) days in the NICU, leaves the NICU within *k*-days. This is conditioned on their baseline covariate information as well as covariate information that is obtained for these patients up to and including (*s*) days after their admission to the NICU. We model the probability of departure from the NICU within *k*-days from time *t* among patients who have spent (*s*) days in the NICU at time *t* as a function of all available covariate information using the model,

(6) $$g\left[\pi^{\left(k\right)}(Z_{i}^{\left(s\right)},S_{i}=s)\right]=Z_{i}^{\left(s\right)}\beta^{\left(k,s\right)}$$

where ***β***^(*k*,*s*)^ is a vector of model parameters specific to the model above; the superscript *k* reflects the fact that we are modeling the probability of departure from the NICU prior to or on *k* days from some time point and the superscript, *s* denotes that this model is conditional on those who have spent *s* prior days in the NICU. In the above expression, *g*(.) is an appropriate link function (i.e. logit, probit, complementary log-log, etc. since *Y*^(*k*,*s*)^ is binary). Thus, ${\hat{\pi }}^{\left(k\right)}(Z_{i}^{\left(s\right)},S_{i}=s)$ represents the estimated probability of departing the NICU prior to or on *k* days from time (*t*) for the *i*^*th*^ patient who has spent *s* days in the NICU.

#### Predicting the number of departures

An estimate of the expected number of departures at time (*t*+*k*) from time (*t*) among patients who have spent (*s*) prior days in the NICU at time (*t*) is obtained by summing the predicted responses for each patient,

(7) $$\hat{D}\{(t+k);S=s\}=\overset{H_{s}(t)}{\underset{i=1}{\sum }}{\hat{Y}}_{i}^{\left(k,s\right)}$$

where *H*_*s*_(*t*) denotes the number of patients at time (*t*) who have spent (*s*) days in the NICU and ${\hat{Y}}_{i}^{\left(k,s\right)}$ is the predicted response for patient *i*, which can be taken as: 

1. ${\hat{Y}}_{i}^{\left(k,s\right)}$ = ${\hat{\pi }}^{\left(k\right)}(Z_{i}^{\left(s\right)},S_{i}=s)$ or,

2. ${\hat{Y}}_{i}^{\left(k,s\right)}$ is a sample from a $\text{Bernoulli}({\hat{\pi }}^{\left(k\right)}(Z_{i}^{\left(s\right)},S_{i}=s))$.

We opted for the later as it better aligns with the ensemble-based framework described in Section “Ensemble-based forecasting and prediction intervals for census forecasts”. Since the model in equation (7) only considers patients that have spent (*s*) days in the census at time (*t*), *s*_*i*_=*s* for all *i*. Based on the proposed framework, the total number of expected departures *k* days from time (*t*) among *N* patients residing in the NICU at time (*t*), is the sum of the expected number of departures for each *S*=0,1,…,*R* where *R*= max(*S*_*i*_), for *i*=1,2,…,*N* at time (*t*),

(8) $$\hat{D}\{(t+k)\}=\overset{R}{\underset{s=0}{\sum }}\hat{D}\{(t+k);S=s\}$$

Due to sparseness of data for large *R* and also model feasibility, we propose setting *R* to a fixed value that remains constant across forecasts at different times. In summary, there are two main stages in our approach to predicting the number of departures among *N* patients residing in the census at time *t*. In the first stage, we predict the number of departures among patients who have spent *S* days in the census, where *S*={0,1,…,*R*} using model (7) and in the second stage we sum over all predictions obtained in the first stage to obtain an estimate of the expected number of departures from the census.

It is important to note that formula (8) provides an estimate of the expected number of departures *k*-days from time *t* only among the *N* patients residing in the census at time *t*. Given that the number of departures at time *k*-days from time *t* also depends on subjects that arrive between times *t* and *t*+*k*, reliable estimates of the number of departures must also consider these subjects. Since these subjects are not directly observed, we hereafter refer to them as *pseudo-subjects*.

We estimate the probability of departure *k*-days from time *t* for each *pseudo-subject* by taking a random sample - with replacement - of baseline covariates from our available data, $x_{i}^{⋆}$. Using $x_{i}^{⋆}$, we compute the probability of departure, ${\hat{\pi }}^{\left(k\right)}(x_{i}^{*},s_{i}=s)$, prior to or on day (*t*+*k*), for the *i*^*th*^*pseudo-subject*. Putting this into a broader context, if we predict the number of arrivals on day *t*+1 to be ${\hat{\mu }}_{t+1}$, that is $\hat{A}(t+1)={\hat{\mu }}_{t+1}$, then we sample ${\hat{\mu }}_{t+1}$ observations, with replacement, from our available data and use the baseline covariates from those ${\hat{\mu }}_{t+1}$ observations to predict the number of departures prior to or on day *t*+*k* from that group. This process is repeated for day *t*+2 up to day *t*+*k*−1, with $\hat{A}(t+2),\ldots ,\hat{A}(t+k-1)$ forming the basis of how many *psuedo-subjects* are considered at each time point.

As noticed, forecasting census counts becomes increasingly more complex with increasing levels of uncertainty as one considers longer forecasts. While the above examples present the census forecasts as point predictions, it is often the case that better prediction performance can be achieved by summarizing over an ensemble of such forecasts (i.e. mean or median over the ensemble) [16]. In the section that follows, we present an ensemble-based method that can be simultaneously used to (*i*) obtain more reliable census forecasts and (*ii*) obtain prediction intervals for our census forecasts.

### Ensemble-based forecasting and prediction intervals for census forecasts

To generate a representative sample of the possible future states of the census, we propose an ensemble-based procedure for obtaining census forecasts and note that this procedure can also be used to construct prediction intervals. As noted in Remark 1, this approach assumes independence between the number of arrivals *A*(*t*) and the number of departures *D*(*t*) for all *t*=1,2,…,*T*. To illustrate the procedure, recall that *A*(*t*)∼*Poisson*(*μ*_*t*_), where

$$\mu_{t}=\exp\left[\beta_{0}+\phi \cos\left(\frac{2\mathrm{\pi t\omega}}{T}\right)+\overset{\dot{p}}{\underset{i=1}{\sum }}\beta_{i}A\left(t,-,i\right)\right]$$

Let, $\lambda =\;{\left[\beta_{0},\beta_{1},\ldots ,\beta_{\dot{p}},\phi \right]}^{T}$, it follows that $\hat{\lambda }\overset{.}{∼}\text{MVN}(\lambda ,V_{\lambda })$, where $\hat{\lambda }$ represents the maximum likelihood estimate of ***λ*** and **V**_*λ*_ is the inverse of the Fisher Information matrix for ***λ***. Additionally, recall that $D(t+k)=\overset{R}{\underset{s=0}{\sum }}D\{(t+k);S=s\}$, where $D\{(t+k);S=s\}=\overset{H_{s}(t)}{\underset{i=1}{\sum }}Y_{i}^{\left(k,s\right)}$. We have that $Y_{i}^{\left(k,s\right)}∼\text{Bernoulli}(\pi^{\left(k\right)}(Z_{i}^{\left(s\right)},S_{i}=s))$, where:

(9) $$\pi^{\left(k\right)}(Z_{i}^{\left(s\right)},S_{i}=s)=\frac{\exp\left\{\overset{\left(s\right)}{\underset{i}{Z}}\beta^{\left(k,s\right)}\right\}}{1+\exp\left\{\overset{\left(s\right)}{\underset{i}{Z}}\beta^{\left(k,s\right)}\right\}}$$

without loss of generality, assuming a logistic regression model for the probability of departure. It follows then that ${\hat{\beta }}^{\left(k,s\right)}\overset{.}{∼}\text{MVN}(\beta^{\left(k,s\right)},V_{\beta^{\left(k,s\right)}})$, where ${\hat{\beta }}^{\left(k,s\right)}$ represents the maximum likelihood estimate of ***β***^(*k*,*s*)^ and $V_{\beta^{\left(k,s\right)}}$ represents the inverse Fisher Information matrix for ***β***^(*k*,*s*)^.

Recall that a point estimate of census at time (*t*+*k*) from time (*t*) is obtained using equation (2). Thus, we generate an ensemble of predictions of *C*(*t*+*k*), denoted ${\hat{C}}^{\left(r\right)}(t+k)$, where:

(10) $${\hat{C}}^{\left(r\right)}(t+k)=C(t)+\overset{k}{\underset{j=1}{\sum }}{\hat{A}}^{\left(r\right)}(t+j)-\overset{k}{\underset{j=1}{\sum }}{\hat{D}}^{\left(r\right)}(t+j)$$

and *r* denotes a single realization. More precisely, we obtain ${\hat{C}}^{\left(r\right)}(t+k)$ using the following procedure:

For*r*=1,2,…,*M*

1. Denote ***λ***^(*r*)^ as a sample from a Multivariate normal distribution with mean $\hat{\lambda }$ and variance-covariance ${\hat{V}}_{\lambda }$ and ***β***^(*r*)(*k*,*s*)^ as a sample from a Multivariate normal distribution with mean ${\hat{\beta }}^{\left(k,s\right)}$ and variance-covariance ${\hat{V}}_{\beta^{\left(k,s\right)}}$, for *s*=1,2,…,*R*.

2. Conditional on ***λ***^(*r*)^ and ***β***^(*r*)(*k*,*s*)^, compute ${\hat{\mu }}_{t+j}^{\left(r\right)}=\mu (\lambda^{\left(r\right)})$ and ${\hat{\pi }}_{i}^{\left(r)(k\right)}=\pi^{\left(k\right)}(z_{i}^{\left(s\right)},s_{i}=s)|\beta^{\left(r)(k,s\right)})$ for *j*=1,2,…,*k*, *i*=1,2,…*H*_*s*_(*t*), and *s*=1,2,…,*R*.

3. Based on ${\hat{\mu }}_{t+j}^{\left(r\right)}$ and ${\hat{\pi }}_{i}^{\left(r)(k\right)}$, obtain samples ${\hat{A}}^{\left(r\right)}(t+j)$ and ${\hat{Y}}_{i}^{\left(r)(k,s\right)}$ from a $\text{Poisson}({\hat{\mu }}_{t+j}^{\left(r\right)})$ and $\text{Bernoulli}({\hat{\pi }}_{i}^{\left(r)(k\right)})$ respectively, for *j*=1,2,…,*k*, *i*=1,2,…*H*_*s*_(*t*), and *s*=1,2,…,*R*.

4. Based on ${\hat{A}}^{\left(r\right)}(t+j)$, *j*=1,2,…,*k*−1 sample ${\hat{Y}}_{i}^{⋆(r)(k-j,0)}$ for each of the pseudo-subjects.

5. Compute ${\hat{D}}^{\left(r\right)}(t+j)$, for *j*=1,2,…,*k*.

6. Conditional on ${\hat{A}}^{\left(r\right)}(t+j)$ and ${\hat{D}}^{\left(r\right)}(t+j)$, for *j*=1,2,…,*k*, Compute ${\hat{C}}^{\left(r\right)}(t+k)$.

End

where *M* is an upper bound pre-specified by the use (i.e. *M*=1000). Our census forecasts are then obtained by summarizing over the ensemble of predictions, ${\hat{C}}^{\left(r\right)}(t+k),r=1,2,\ldots M$, using for instance, the mean or median. Furthermore, this approach can also be used for the construction of 95% prediction intervals by computing the associated percentiles of the ensemble of predictions.

Up to now, we have considered the components of the census forecasting model, namely the arrivals and the departures, as separate entities. To provide further intuition, we provide an example of our proposed census forecasting model in Additional file 1.

## Results

Our census forecasting model was applied to data collected by the third author, a neonatologist at the Department of Pediatrics, Women and Infants Hospital, 101 Dudley St., Providence, RI with the goal of obtaining accurate short-term census forecasts. Women & Infants Hospital, Department of Pediatrics maintains several patient databases designed for quality monitoring. One of these, the *Risk-Adjusted Length of Stay Database* has prospectively collected data on neonatal severity of illness in the first week of life. Maintained meticulously from April 2008 through June 2010, it includes 2660 consecutive admissions that stayed in the NICU greater than 24 hours. It includes fields for patient level information, such as: demographic data, hospital stay, and severity of illness indices.

The patient level data used for this analysis consisted of 1001 consecutive NICU admissions, born between April 1, 2008 through March 31, 2009, that had complete data at the time of analysis. With IRB approval, the medical records of all newborns born between April 1, 2008 through March 31, 2009 admitted to the NICU were obtained retrospectively for data extraction. All newborns admitted to the NICU at Women and Infants Hospital within 24 hours of birth were recruited and ranged in morbidity from minimal to sever. Newborns were excluded if they (*i*) died prior to NICU admission; (*ii*) were admitted for pre-terminal comfort care (defined as neither intubation nor cardiorespiratory resuscitation); (*iii*) had a major congenital anomaly. All procedures and study materials were approved by the Institutional Review Board at Women & Infants Hospital, Providence RI.

Numerous illness severity indices have been developed for predicting mortality in the neonatal population. Two of these, the Score for Neonatal Acute Physiology, Perinatal Extension (SNAPPE) [17], and Morbidity Assessment Index for Newborns (MAIN) [18], have been shown to be promising predictors for an individual’s length of stay in the NICU, even after adjusting for an individuals birth weight and gestational age [15]. SNAPPE, scored from 9 categorical variables which are acquired in the first few days of life, provides an overview of overall physiologic health in the neonate. MAIN is scored from an accumulation of 47 morbidities in the first week of life and similar to SNAPPE, provides an assessment of overall neonatal physiological health. Of particular importance to the forecasting models we propose is that both SNAPPE and MAIN scores are collected at two time points during the perinatal period. Specifically, SNAPPE scores are collected on day 1 and day 3 of life, whereas MAIN scores are collected on day 1 and day 7 of life. We refer to the SNAPPE scores collected on day 1 and day 3 of life as SNAPDOL1 and SNAPDOL3. Similarly, we refer to the MAIN scores collect on day 1 and day 7 of life as MAIN1 and MAIN7 respectively. In addition to MAIN and SNAPPE, both gestational age and birth weight, obtained upon admission to the NICU, were used in our forecasting models. The gestational age was taken as actual, completed gestational weeks. An infant who was 32 6/7 was recorded as 32 weeks. An infant who was 33 0/7 was recorded as 33 weeks. Gestational age was the best obstetrical estimate. If that was not available, it was followed by the pediatric assessment. Birth weight was obtained by documentation in the operating room, delivery room or NICU, whichever was recorded first. See Table 1 for a summary of the patient level data.

**Table 1:** Summary statistics for the patient level data (N = 1001)

**Variable**	**Variable type**	**Mean**	**Median**	**Standard deviation**	**Range**
LOS	Discrete	20.1	10.0	25.4	(1,227)
BWEIGHT	Continuous	2418.0	2335.0	939.8	(360, 5493)
GESTAGE	Continuous	34.6	34.0	4.0	(23, 42)
SNAPDOL1	Discrete	8.2	0.0	13.1	(0, 103)
SNAPDOL3	Discrete	5.0	0.0	10.4	(0, 86)
MAIN1	Continuous	484.8	417.0	314.0	(0, 2139)
MAIN7	Continuous	661.2	607.0	361.3	(0, 2486)

LOS represents a patient’s length of stay in the NICU, BWEIGHT represents a patient’s birth weight in grams, GESTAGE denotes a patient’s gestational age in weeks, SNAPDOL1 represents a patient’s SNAPPE score recorded after 1 day spent in the NICU, SNAPDOL3 represents a patient’s SNAPPE score recorded after 3 days spent in the NICU, MAIN1 represents a patient’s MAIN score recorded after 1 day spent in the NICU and MAIN7 represents a patients MAIN score recorded after 7 days spent in the NICU. The smaller the SNAPPE and MAIN scores, the physiologically healthier the child.

Arrival data used in the development of the census forecasting models consisted of the number of daily admissions to the NICU at Women and Infants hospital from January 1, 2008 through May 20, 2009. Collectively, both the patient level data as well as the arrival data were used to construct a NICU census forecasting model for predicting the census counts at 1, 3, 5, and 7 days in advance. All analyses were carried out using R version 2.11.

### Fitting the seasonality adjusted PAR model for predicting arrivals

We first estimated the autocorrelation function (ACF) to determine the presence of seasonality and weekly trends in our data. We observed a modest semi-annual trend in our data with peaks in the number of arrivals in the spring and fall months. Since our data consists of approximately a year and a half of daily admissions counts, our empirical estimate of the frequency of the seasonality trend was ($\hat{\omega }=3$), which constitutes 1 cycle per 26 weeks. We used the the Bayesian Information Criterion (BIC) [14] to determine the optimal order, ($\dot{p}$) of our PAR models. More specifically, we adjusted for seasonality and fit several PAR(*p*) models varying the order (*p*) and selected the model that resulted in the lowest BIC. Based on this approach, we estimated the optimal order to be ($\dot{p}=7$). Fitting an order (7) seasonality adjusted PAR model, we computed within sample predictions and found the mean-squared error (MSE) to be 3.48, which is marginally better than using predictions based on the estimated mean number of daily admissions (MSE = 3.55).

### Predicting departures

**Figure 2:**
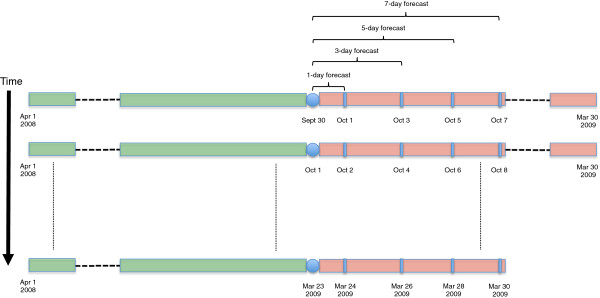
**Diagram illustrating the continuously updating forecasting procedure.** Diagram illustrating the continuously updating forecasting procedure for prospectively forecasting the NICU census data described in the data application. The training and testing sets are denoted by the green and red boxes, respectively. Initially the training data consisted of patients who were admitted to the NICU between April 1st, 2008 to September 30, 2008. As each day progresses (increasing time), the training data absorbs part of the testing data.

As previously described, the patient level data used in the development of our forecasting models consisted of 1001 consecutive NICU admissions from April 1st 2008 through March 30th 2009. In order to validate our forecasting model, we split the patient level dataset into training and testing sets. The *initial* training data consisted of 598 patients who were admitted to the NICU from April 1st 2008 up to and including September 30th 2008. Furthermore, our testing data consisted of 603 patients who were admitted to the NICU from October 1st 2008 up to and including March 30th 2009. We used a realistic *continuously updating* (Figure 2) forecasting procedure for prospectively forecasting the NICU census as this approach most closely mimics how one would use these forecasting models in practice. For example, consider forecasting the NICU census on October 1st 2008 from September 30th 2008, the first and last date in our testing and training data, respectively. To forecast the NICU census on this date we used all available training data to develop our prediction models for departure. We then constructed the current NICU census on September 30th 2008, which consisted of patients who arrived to the NICU prior to or on September 30th and departed after September 30th. Using the current census count on September 30th, our predicted number of arrivals on October 1st, and the predicted number of departures from the census on October 1st, we were able to obtain an estimate of the census count on October 1st using the relationship between the current census count, arrival counts, and departure counts as described in equation (2). We continue this procedure to obtain estimates of the census counts up to and including March 30th 2009, the last date in our testing data. As noted, a desirable feature of our *continuously updating* procedure is that the training data is conditional upon date and thus more information is available for model development at each sequential date. As pointed out, this most accurately reflects how one would use our forecasting models in a practical application.

We considered conditional logistic repression models to obtain an estimate for the expected number departures. In order to obtain prospective census forecasts at 1, 3, 5, and 7 days in advance, we fit a series of conditional logistic regression models which were collectively used to obtain an estimate of the number of departures. In particular, the logistic regression models that were used to inform the expected number of departures were stratified by the number of days a patient occupied NICU, which in turn had implications on what covariate information was available for those patients. For instance, at baseline, the only covariate information for a patient was their birth weight and gestational age. However SNAPDOL1 and MAIN1 scores were available for patients who occupied the NICU for at least one day. Furthermore, SNAPDOL3 and MAIN7 scores were available for patients who occupied the NICU for at least 3 and 7 days respectively. As mentioned, the logistic regression models that were used to inform the expected number of departures were stratified by the number of days a patient occupied NICU, such that *S*∈{1,2,…,≥10}, where *S* represents the number of days a patient had occupied the NICU at some time point. The upper bound for *S* was selected as such due to concerns regarding data spareness as well for computational and model feasibility. Given the high volume of models this framework requires, results from our individual model fits are omitted, but available upon request.

### Predicting census

**Figure 3:**
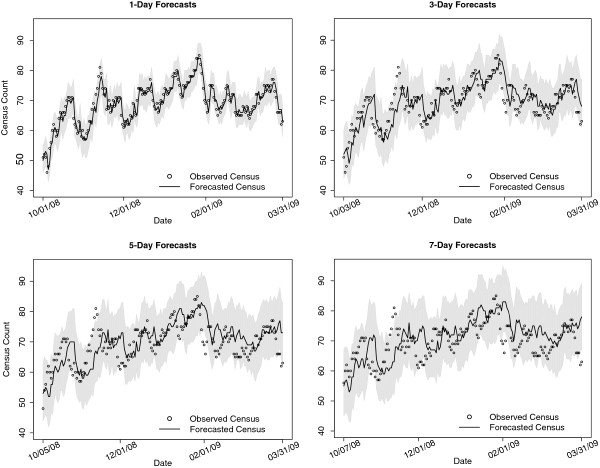
**Plots of the observed and predicted census.** Plots of the observed and predicted census with 95% prediction intervals. Predictions are based on data observed up to the date being predicted.

**Figure 4:**
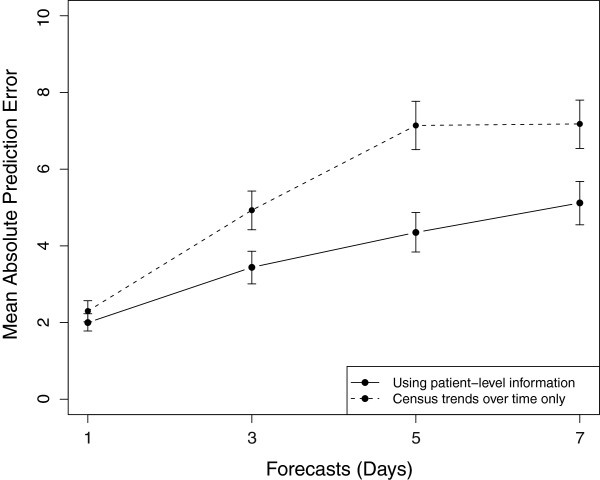
**Plot of the Mean Absolute Prediction Error (MAPE).** Plot of the Mean Absolute Prediction Error (MAPE) and corresponding 95% CIs for 1, 3, 5, and 7 day forecasts among our proposed model (denoted by points along the solid line) and a model that ignores patient-level information (denoted by points along the dashed line).

In Figure 3 we present the results of the observed and forecasted census counts among the testing data for 1, 3, 5, and 7 day forecasts. The forecasted census counts were obtained by taking the median of an ensemble of 1000 census predictions for each day in our testing data. As noted from Figure 3, the 1, 3, 5, and 7 day forecasts appear to capture the global trends in observed census counts, however as expected, locally the accuracy of our census forecasts diminishes as a function of the length of forecasts. A plot of the residuals (observed - forecasted census; figure not shown) shows a moderate tendency of our forecasting model to overestimate the census. This overestimation however, becomes more pronounced for the longer forecasts. The mean absolute prediction error (MAPE) for 1, 3, 5, and 7 day forecasts were estimated to be 2.00, 3.44, 4.35, and 5.12 respectively (Figure 4). Moreover, we estimated that 98%, 94%, 90%, and 91% of observed census counts were within the 95% prediction intervals for the forecasted census.

**Figure 5:**
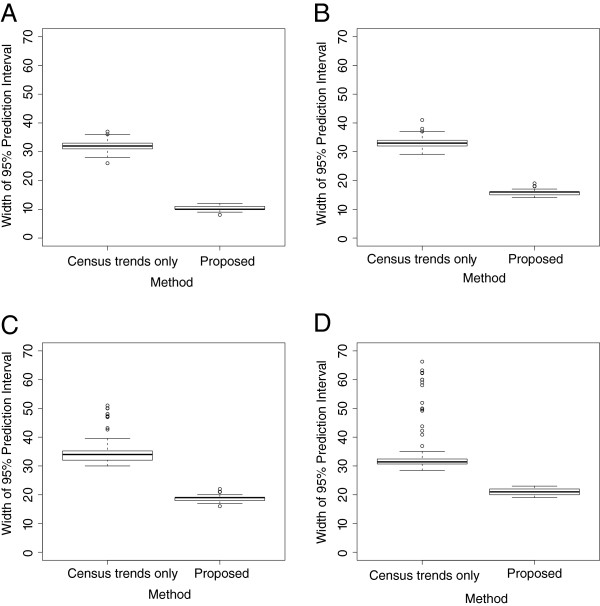
**Boxplots of the widths of the 95% prediction intervals.** Boxplots of the widths of the 95% prediction intervals among the proposed forecasting method and a method that models census trends over time only, for (**A**) 1-day forecasts, (**B**) 3-day forecasts, (**C**) 5-day forecasts, and (**D**) 7-day forecasts.

We also compared the results of our forecasting model to the results of an approach that used only census counts over time and thus ignored available patient-specific information. Similar to our methodology for predicting the number of arrivals, this method utilized a seasonality adjusted PAR model, where the seasonality frequency (*ω*) of was determined empirically using the ACF and the order (*p*) of the PAR model was determined using the BIC. Using the census counts for our training data, we estimated the seasonality frequency and the optimal order to be $\hat{\omega }=1$ and $\dot{p}=5$ respectively. Fitting a seasonality adjusted PAR(5) model to the census counts for our training data and forecasting the NICU census for our testing data, we determined the MAPE for 1, 3, 5, and 7 day forecasts to be 2.30, 4.93, 7.14, and 7.18 respectively (Figure 4). Moreover, we estimated that the coverage probabilities for this approach (i.e. percent of observed census counts that were within the 95% prediction intervals) were nearly 1 for 1, 3, 5, and 7 day forecasts, however the width of the 95% predictions intervals were substantially larger for this method compared to our proposed forecasting method (Figure 5).

## Discussion

In this paper we presented a novel methodology for forecasting the census in a Neonatal Intensive Care Unit (NICU). Our model explicitly incorporates both arrival trends over time in the NICU and patient-level clinical information. The census forecast is computed from the current census count, predicted arrivals and predicted departures. We proposed an ensemble-based procedure for obtaining census predictions that used seasonality adjusted PAR model to model arrival trends in the NICU and stratified conditional logistic regression models incorporating baseline and time-dependent covariates for modeling the probability of departure. Our model is efficient because it integrates arrival trends over time as well as patient level information.

We note that the ideal data set to train and test our forecasting model is one that consists of several years worth of patient-level and arrival data. The former is crucial to the development of accurate and reliable models for predicting the probability departure, while the latter is integral to attainment of a model that can predict the number of census arrivals with a high degree of accuracy. The arrival data used in this analysis consisted of only approximately a year and a half of admission information, thus we are limited in our ability to ascertain long-term seasonality trends. Our finding of a half-year periodicity in arrivals to the NICU (peaks in the spring and fall months) is consistent with numerous studies, which have reported seasonality trends in delivery rates [19-22]. Plausible explanations for this finding include temperature or photoperiod (affecting hormonal concentrations, sperm quality or sexual activity), seasonal variation in pregnancy loss, or cultural factors [23]. Despite the evidence for seasonal patterns in delivery rates, the nature of seasonality effects tend to vary across different racial and ethnic groups, maternal education levels, and marital status of the underlying population [22]. Since the PAR model described in Section “Predicting the number of arrivals” can be generalized to account for *K*-many seasonality effects using the sum of both cosine and sine terms with different frequencies, differing arrivals patterns across different study populations can be easily accommodated under this general framework.

The arrival data used in this analysis and perhaps the nature of NICU arrival data in general, presented challenges in the formulation of an efficient and reliable model for predicting the number of NICU admissions. This feature is a likely candidate to explain the modest overestimation that was observed in the validation of our forecasting model, which became more pronounced for increasing lengths of forecasts. The primary reason for this is that census forecasts beyond 1-day rely heavily on accurate predictions of the number of arrivals for intermediary days, thus bias in intermediary predictions can give rise to considerable under- over-estimation in downstream census forecasts. We note that the observed tendency of our proposed approach to overestimate the census, particularly at the later time-points (i.e., 5- and 7-day forecasts), could be used to recalibrate our census forecasting procedure in a manner similar to [24]; however, arrival, census, and patient-level data over a longer span of time than what were used here would be needed to effectively implement such an approach.

Our justification for using a conditional logistic regression framework for predicting the number of departures was motivated by two principle issues. As a result of our general forecasting framework, our interest was primarily focused on the expected number of departures for a cohort of patients currently residing in the census. Thus, treating each patient within a cohort as independent, the expected number of departures for a given cohort can be efficiently estimated by summing the individual predictions for departure for each patient. The idea of predicting the probabilities of departure as opposed to length of stay predictions lends itself nicely to a logistic regression framework. An alternative approach involves using length-of-stay distributions within a queueing theory analysis. However, unlike the framework described here, such an approach would not facilitate the attainment of the subject-specific probabilities of departure, which is of interest to clinicians. Secondly, it is also of clinical interest to have estimates of the probability that a patient leaves the NICU some *k* days into the future, which can be conveniently extracted using our proposed approach. While our approach was based on predicting the probability of “healthy-discharge” from the NICU, “non-healthy discharge” (Pediatric Intensive Care Unit (PICU or death in the NICU) represented a relatively small proportion of the study population considered here (3% and 2%, respectively). For this reason and because including such subjects would necessitate a framework that simultaneously models the probability of leaving the NICU in a variety of different ways (i.e., health discharge, PICU, death), adding considerable statistical and computational and complexity, we opted to exclude these subjects for the development and validation of our proposed methodology.

The results obtained from validating our forecasting model demonstrated that the ability to accurately forecast the NICU census was largely a function of the length of forecasts, a common feature of nearly all forecasting models. Furthermore, the estimated MAPE and correlations among the predicted and observed census between our forecasting model and an approach based solely on census trends over time, suggest that incorporating patient-specific information has the capacity to improve census predictions, especially for the longer forecasts (i.e 3, 5, and 7 day forecasts). Additionally, the narrower widths of the 95% prediction intervals for our forecasting model relative to the forecasting model based only on census trends over time, suggest that more precise predictions can be obtained using our forecasting model.

## Conclusions

In summary, census forecasting models that utilize (*i*) arrival trends over time and (*ii*) patient-specific baseline and time-varying information make the most of data which is typically available in the NICU and as demonstrated, have the potential to be a useful tool for prospectively estimating the NICU census.

## Abbreviations

NICU: Neonatal intensive care unit; PAR: Poisson autoregressive; MAPE: Mean absolute prediction error; MSE: Mean squared error; LOS: Length of stay.

## Competing interests

The authors have no competing interests to report.

## Authors’ contributions

DK and HO conceived of the statistical methodology and participated in the drafting of the manuscript. DK also performed the statistical analysis. JB participated in the design and coordination and also helped to draft the manuscript. All authors read and approved the final manuscript.

## Pre-publication history

The pre-publication history for this paper can be accessed here:

http://www.biomedcentral.com/1471-2288/13/67/prepub

## Supplementary Material

Additional file 1 **An example of the census forecasting model.pdf.** An illustration of the proposed forecasting model using a practical example. Click here for file
