# Supplementary material for: Ensemble-based methods for forecasting census in hospital units
Source: BMC Med Res Methodol. 2013 May 30;13:67. doi: 10.1186/1471-2288-13-67 (PMC3680345; doi:10.1186/1471-2288-13-67)
Supplement: Additional file 1 — An example of the census forecasting model.pdf. An illustration of the proposed forecasting model using a practical example. [file 1471-2288-13-67-S1.PDF]

Ensemble-based Methods for Forecasting Census in Hospital Units

Devin C. Koestler*[1], Jesse Bender[2] and Hernando Ombao[3]

[1]Department of Community and Family Medicine, Geisel School of Medicine at Dartmouth College, Lebanon NH
[2]Department of Statistics, University of California at Irvine, Irvine, CA 92697, USA
[3]Department of Pediatrics, Women and Infantsospital of Rhode Island, Providence, RI 02905, USA

Email: Devin C. Koestler *- devin.c.koestler@dartmouth.edu; Jesse Bender - gbender@wihri.org; Hernando Ombao - hombao@uci.edu;

*Corresponding author

1 Additional File 1 - An example of the census forecasting model

To provide some intuition into how the census forecasting model functions, we consider the examples below. For the purpose of simplicity, the census predictions given in the succeeding examples are based on point predictions of the census.

1. Suppose that on Day '0' there are no patients in the NICU from the previous day. Further suppose that there are 6 new arrivals to the NICU on this day. Hence using our previous notation; $H_0(0) = A(0) = C(0) = 6$.

2. On Day '1', suppose that there are 3 new arrivals, hence $H_0(1) = A(1) = 3$. Furthermore, suppose that 4 out of the 6 patients that arrived on Day '0' are still in the NICU, $H_1(1) = 4$. That is, among the 6 arrivals from the previous day, 2 patients were discharged from the NICU between Day '0' and Day '1'. Thus, the census count on Day '1' is, $C(1) = C(0) + A(1) - D(1) = 6 + 3 - 2 = 7$.

3. From Day '1', we want to estimate the expected census count on Day '2'. Recall: $\widehat{C}(2) = C(1) + \widehat{A}(2) - \widehat{D}(2)$. In this scenario, $C(1) = 7$ is known. Since we are forecasting from Day '1', an estimate of the expected number of arrivals on Day '2' is obtained via prediction from our PAR model, that is; $\widehat{A}(2)$ is a sample from a Poisson distribution with mean $\widehat{\mu}_2 = \widehat{\beta_0} + \widehat{\beta}_1 A(1)$. For the purposes of this example we assume that there are are no seasonality trends (ie: $\phi = 0$). An estimate of the number of

departures on Day '2' is given by:

$$\widehat{D}\{(2)\} = \sum_{S=0}^{1} \widehat{D}\{(2; S = s)\} = \sum_{i=1}^{H_0(1)} \widehat{Y}_i^{(1,0)} + \sum_{i=1}^{H_1(1)} \widehat{Y}_i^{(1,1)}$$

where $H_0(1) = 3$ the number of patients who have spent '0' days in the NICU at Day '1' (i.e. the number of children admitted to the NICU on Day '1') and $H_1(1) = 4$ the number of patients who have spent '1' day in the NICU at Day '1' (i.e. the number of children who were admitted to the NICU at Day '0' and still remain in the NICU on Day '1').

In the above, $\widehat{Y}_i^{(1,0)}$ is a sample from a Bernoulli distribution with probability $\widehat{\pi}^{(1)}(\mathbf{x}_i, s_i = 0)$ and $\widehat{Y}_i^{(1,1)}$ is a sample from a Bernoulli distribution with probability $\widehat{\pi}^{(1)}(\mathbf{z}_i, s_i = 1)$, where $\widehat{\pi}^{(1)}(\mathbf{x}_i, s_i = 0)$ and $\widehat{\pi}^{(1)}(\mathbf{z}_i, s_i = 1)$ are estimated from the data. Take note of the two sums that define our estimate of the number of departures on Day '2'. The first sum represent the expected number of departures among the group that was admitted to the NICU on Day '1' and the predicted probabilities of departing the NICU for each patient in this group are based only on baseline covariates since those are the only covariates we have for those patients at that time. The second sum represents the expected number of departures among the group that was admitted to the NICU on Day '0', but still remained in the NICU on Day '1'. Since these patients have spent one full day in the NICU, the predicted probabilities of departing the census for each patient in this group are based on both their baseline covariates as well as any additional covariate information that was collected during their first day in the NICU.

4. Now suppose that instead of forecasting the census on Day '2' from Day '1' we are interested in forecasting the census on Day 'k' from Day '1'. Again using our previously defined framework, our estimate of the census on Day 'k' from Day '1' is given by: $\widehat{C}(k) = C(1) + \sum_{i=2}^{k} \widehat{A}(i) - \widehat{D}\{(k)\}$, where $C(1) = 7$, as previously defined and $\widehat{A}(i)$, $i = 2, 3, \ldots, k$, represents our estimates of the number of arrivals from Day '2' up to Day 'k', which we predict via the estimates from our PAR(p) model. $\widehat{D}\{(k)\}$ represents our estimate of the number of departures among the cohort of patients currently occupying census as well as patients that are admitted to the census between Day '2' and Day 'k'. In other words,

$$\widehat{D}\{(k)\} = \sum_{j=2}^{k-1} \sum_{i=1}^{\widehat{A}(j)} \widehat{Y}_i^{\star(k-j,0)} + \sum_{i=1}^{H_0(1)} \widehat{Y}_i^{(k-1,0)} + \sum_{i=1}^{H_1(1)} \widehat{Y}^{(k-1,1)}$$

In the formulation above, $\sum_{j=2}^{k-1} \sum_{i=1}^{\hat{A}(j)} \widehat{Y}_i^{\star(k-j,0)}$, where $\widehat{Y}_i^{\star(k-j,0)}$ is a sample from a Bernoulli distribution with parameter $\widehat{\pi}^{(k-j)}(\mathbf{x}_i^{\star}; s_i = 0)$, represents the estimate of the expected number of departures among the group of patients who are admitted to the census between Day '2' and Day '$(k-1)$'.

Since these subjects are not directly observed, we refer to these subjects as *pseudo-subjects.* Section 2.3 of the manuscript text describes our approach for estimating the probabilities of departure for *pseudo-subjects.*
